# School Factors Strongly Impact Transgender and Non-Binary Youths’ Well-Being

**DOI:** 10.3390/children9101520

**Published:** 2022-10-04

**Authors:** Janie Kelley, Annie Pullen Sansfaçon, Morgane A. Gelly, Lyne Chiniara, Nicholas Chadi

**Affiliations:** 1 Faculty of Medicine, Department of Medicine, University of Montreal, Montreal, QC H3C 3J7, Canada; 2 School of Social Work, University of Montreal and Centre de Recherche en Santé Publique, Montreal, QC H2Y 2G7, Canada; 3 Division of Endocrinology, Department of Pediatrics, Saint-Justine University Hospital Centre, University of Montreal, Montreal, QC H2Y 2G7, Canada; 4 Division of Adolescent Medicine, Department of Pediatrics, Sainte-Justine University Hospital Center, University of Montreal, Montreal, QC H2Y 2G7, Canada

**Keywords:** students, school, transgender, non-binary, well-being

## Abstract

Background: School plays an important role in transgender and non-binary (TNB) youths’ life and well-being. The aim of this study was to gain a better understanding of how the lived experiences, gender affirmation and challenges encountered by TNB youths in the school setting affect their well-being. Method: Our study was a qualitative secondary data analysis, based on the interviews of 12 Canadian TNB youths aged 15–17 years old. Results: We found that TNB students’ well-being was closely related to the acknowledgment of gender identity at school. Several factors, including school socio-cultural environment, teachers’ and peers’ attitudes and behaviours, school physical environments and the respect of confidentiality of gender identity were all found to impact TNB students’ well-being. To face adversity related to some of these factors, TNB youths used several contextually driven strategies such as compromising, educating, and sensitizing others about gender diversity and avoiding certain people or situations. Conclusion: Our results highlight the important influence of school climate and culture, as well as teachers’, school personnel’s and peers’ behaviours and attitudes on TNB youths’ well-being. Our findings can guide future interventions to help schools become more inclusive and supportive of gender diversity.

## 1. Introduction

Gender minority youths experience high rates of adverse mental health outcomes during early adolescence [1]. Compared to cisgender youths, transgender and non-binary (TNB) youths are more likely to report feeling unhappy and isolated [2] and to report depressive symptoms [3]. TNB youth are at higher risk of engaging in harmful behaviours such as cigarette, alcohol, and cannabis use [2]. Additionally, they are more likely to disclose suicidal ideation and attempts and non-suicidal self-injuries than their cis-identifying peers [2,4].

The mental health and well-being of individuals belonging to minority groups, including gender minorities are strongly related to social environmental factors [5,6]. Yet, because of the dominance of hetero and cisnormative ‘norm’ in schools [7,8], the climate may often be unwelcoming to gender-minority youths [9,10]. This constitutes a significant concern given that school is where a major part of teenagers’ daily activities and social interactions take place. Thus, school climate has an important effect on students’ well-being [11,12].

Existing literature describing TNB youths’ experiences in schools often fails to distinguish TNB teenagers from their lesbian, gay and bisexual (LGB), cis-identifying peers. However, TNB young people experience challenges at school that are different from those encountered by their non-heterosexual, cisgender peers [2,13]. Thus, TNB students’ experiences at school remains underexplored. 

Feeling safe at school is a major concern for many TNB youth according to cross-sectional studies [13,14], and up to 36% of Canadian TNB youth report feeling unsafe [14]. Partly because of safety issues, TNB students are more likely to skip school, [12,13,15]. Certain physical environments at school are identified as feeling more unsafe such as bathrooms and locker rooms [13,16,17]. 

Literature on TNB students, has shown that TNB students are at higher risk of victimization (i.e., being targeted or discriminated by their peers) due to their gender identity [12,18,19]. TNB youth often experience violence, adversity, and trauma at school [20] and are more frequently victims of gender-based bullying at school and online, including verbal and physical attacks [2,13]. Bullying and anti-LGBTQ language and attitudes have been associated with lower self-esteem, lower self-reported school grades and decreased sense of safety at school among transgender youths [17,21]. 

TNB students experiencing higher levels of victimization are less likely to feel supported by an adult at school [22]. In addition, TNB youths report receiving less support from peers and staff members at school than other students [2,4] and they perceive teachers to be less open to gender diversity [23]. As a result, TNB youth are at higher risks of absenteeism and dropping out of school [23].

Most studies focusing on TNB youth well-being at school rely on cross-sectional surveys or other quantitative approaches [24,25]. These methods, on the one hand, while useful to prove or disprove hypotheses and establish correlations, provide limited insights into the broader experiences of TNB youths. Qualitative research, on the other hand, allows to gain deeper, more nuanced and meaningful insights into the experiences of a group that is known to be diverse in itself [26]. For example, conducting interviews or focus groups with TNB youth can help clarify some of the issues they face, but also illustrate some of the protective dimensions that may facilitate their wellbeing and that are often less articulated in large quantitative surveys. Examples provided by youth who participate in interviews can help demonstrate how school experiences are shaped and what schools can put in place to better support their pupils. Investigating TNB youth experience in schools from a different angle therefore has important health, social and policy implications, and could guide school authorities, community organizations, health providers, and decision makers on how best to increase support for TNB youths

Drawing from qualitative data, this article aims to provide a more thorough understanding of one of these factors that emerged as fundamental to youths’ wellbeing: the school environment. To achieve this goal, we will address the three following questions: (1) What are the lived experiences of TNB youths in the school setting, and how are these impacting their well-being and resilience? (2) How is the affirmation of gender identity supported (or undermined) among TNB students in the school setting? (3) What are the strategies employed by TNB students to face adversity and obstacles imposed on them by the school cultural, social and physical environments?

## 2. Materials and Methods

### 2.1. Overarching Study

As part of the larger project ‘Digging Beneath the Surface: An Intersectional Exploration of Trans Youth Experience’, led by the Canada Research Chair on Transgender Children and their Families, and funded by the Canadian Social Sciences and Humanity research Council, the team decided to specifically analyze minor (under the age of 18 years) TNB youth narratives regarding their experience in school settings. The project aimed to identify oppressive factors and structures that negatively influence TNB youths’ well-being, factors that contribute to TNB youths’ resilience and thriving, and to understand how oppression and resistance processes interact. 

This study used a community-based participatory research approach [27] and was conducted in collaboration with local community organizations working specifically to support TNB youths. Data collection took place in two phases between 2016 and 2018, with consultations with TNB youth and broader discussions with community partner representatives between and after data collections.

### 2.2. Participants and Recruitment for the Overarching Study

The project included 54 interviews with 15–25-year-old TNB youths, living in different cities of the province of Québec. Participants were recruited with the help of community partner organizations via paper (posters and flyers) and online invitations. Recruitment efforts aimed to achieve participant diversity related to gender, age, citizenship status, cultural and socioeconomic backgrounds. According to the latest available census data [28], the majority (87%) of Québec population identifies as White/Caucasian with 13% identifying as a visible minority. The most common ethnic origins other than Canadian included European (38.1%), Asian (7.1%), African (4.8%), North American Aboriginal (4.5%) and Latin, Central and South American (2.1%). Written consent was obtained from all participants before participation, and TNB youths were informed that they could withdraw from the study at any point until publication of the data with no impact on any support services provided to them. Participants received CAD 30 compensation in recognition for sharing their experiences.

### 2.3. Design 

Interviews were conducted in person, in a location and language (French or English) chosen by the participant, by an interviewer identifying as TNB. Interviews were audio-recorded, with the participants’ authorization, and lasted approximately two hours. Interviews were transcribed by the interviewer to ensure accuracy of study materials. 

For this article, we selected the transcripts of participants under the age of 18 -years, for a total of twelve participants. This age range was selected to include youth who are currently in high school in Québec. We excluded older participants as their high school experience would not have been as recent and this could have had an impact on the relevance of our analysis. All the interviews included in this analysis were conducted between November 2016 and June 2018 which means that all participants had a high school experience with the same level of legal protection against gender identity related discrimination. Indeed, in June 2016, gender identity and expression became protected by the Quebec Charter of Rights and Freedoms and minors aged 14 years and older gained the civil right to change their gender markers on their birth certificate without parental consent. Hence, to focus on youth who only have a current or very recent experience within this framework of new protection allows us to have a better understanding of current TNB youth experience in high schools. Conversely, including older participants who would have discussed their high school experience retrospectively would have produced an analysis that is less accurate because they did not have the same legal protection than what is available today.

A different pseudonym was chosen for each transcript to preserve participant anonymity. For this paper, the twelve interviews were reanalyzed, with a focus on school experiences and gender affirmation at school, on the way school impacted participants’ well-being and resilience and the strategies participants used when facing obstacles and adversity.

### 2.4. Sample

Among participants, two identified as trans girls, four as trans boys and six as non-binary youth. All participants were White except for one participant who described themselves as being from a mixed heritage. This lack of cultural diversity in our sample is unfortunate as it does do not reflect the full population from which we selected the sample (15–25 years old) which includes youth from diverse cultural backgrounds. However, youth aged between 15 and 17 years were homogenous in terms of cultural background. One participant lived in a rural area while all others lived in large urban cities. All twelve participants mentioned that they had “come out” about their gender identity while in high school. At the time of the interview, nine participants were still in high school while three had recently graduated. Information about where participants went to school was not explicitly collected. However, the fact that youths came from different cities suggests that they also went to different schools. All three college students described their school experiences during the interview, which we drew from for our analysis of high school experience. For those who had transitioned to college at the time of the data collection, we also included some of their insights on strategies (theme 2) to navigate new environments as this seems particularly important and relevant to understand TNB youth experiences at school. 

### 2.5. Data Analysis

Analyses were conducted in accordance with Meyer’s [5] minority stress theory which suggests that stress is not only attributable to personal life events but can also come from a stressful environment. Additional stress resulting from a hostile environment requires higher levels of adaptation in minority groups. Accordingly, it is expected that not only individuals, but school, social and cultural structures themselves could be responsible for higher levels of stress and mental health issues experienced by TNB youths. 

Data were analyzed through Braun and Clark’s thematic analysis [29], which involves searching across a dataset to find repeated patterns or meaning. All authors involved in the analysis and the writing up of the paper have previous experience in conducting research and/or clinical work with trans and non-binary youth. Team members who conducted the coding and the analysis of the data include cis and non-binary identities, as well as a caregiver of a gender-diverse youth. Racial and ethnic identities include White, Middle Eastern and Indigenous identities. Team members also have diverse professional backgrounds (medicine, social work, nursing, and sociology).

Our first step was to familiarize ourselves with the data. To do so, JK conducted a first reading of the non-coded transcripts, originating from the initial study, and retained all sections of the interviews containing school-experiences-related content. Each of these sections were read multiple times. Then, for the second phase of our analysis, the interviews were manually and systematically coded by JK, generating an initial set of codes. In phase 3, codes expressing similar concepts were identified and combined to describe common themes. These groups of codes were discussed with all study members leading to the formation of thematic categories. After discussion with the research team, in phase 4 of our thematic analysis, all extracts and sections of the interviews discussing school issues were reread. Codes were revised to ensure they were exclusive to their category. Then, in phase 5, categories were named and assembled as the themes started to emerge. In phase 6, a final reorganization of codes and categories took place, in parallel with drafting of this manuscript. According to Braun and Clarke [29], writing up is an essential part of the analysis. Considering the non-linear process of thematic analysis, phases 4 and 5 were revisited all through the writing of this manuscript. All study themes were discussed with a non-binary person (MG). Each step of the process was supervised by a co-author experienced in qualitative research (APS) and in working with TNB youths in clinical and non-clinical settings (NS and LC). 

## 3. Results

Youth explained that their wellbeing was mostly conditioned through experiences of validation of their gender identity. Study results will be presented in two sections. First, through the discussion of impeding and protective factors for TNB youth wellbeing, and second, through the description of strategies used by TNB youth to face adversity. 

### 3.1. Theme 1: Obstacles and Protective Factors Impacting TNB Youth Wellbeing 

Most participants provided examples of how their identity was affirmed, ignored, or diminished at school according to three main dimensions, namely school climate and culture, direct behaviours and attitudes of school staff, and behaviours and attitudes of peers. 

#### 3.1.1. School Climate and Culture about Gender Diversity and TNB Students 

School climate and culture was an important dimension influencing TNB students’ identity acknowledgment. A lack of knowledge about TNB youth identities was perceived by study participants as a major obstacle in getting their identity validated and their rights respected by students, teachers, and school personnel. Some participants felt they were schooled in an environment where the lack of understanding of gender diversity was generalized rather than stemming from a limited number of individuals. This was illustrated by Anto, a 17-year-old non-binary student, who mentioned how both students and staff had a limited understanding of gender diversity when they “came out”:

*“When I came out I said, ‘Hey, my name’s [chosen name] now,’ I don’t go by, like, my birth name. People were confused, they didn’t know what it was.”*

(Anto, 17-year-old non-binary student)

Aside from the overall lack of knowledge about gender diversity, participants felt that the over-representation of cisnormativity in school prevented adequate inclusion of TNB youth. Arthur, a 17-year-old non-binary teenager, explained how they were left aside in their gym class when sports teams were separated between boys and girls:

*“So I just sat on the bench. And then the teacher was like ‘Well, go play.’, and I was like ‘Yeah, but what side do I play on?”.* * (Quotations followed by «*» have been translated from French).

(Arthur, 17-year-old non-binary student)

Overall, the lack of awareness and acceptance of gender diversity and the overabundance of gender stereotypes and cis-gendered assumptions at school were identified as very difficult to live with:

*“[…] people will judge that something is feminine then they’ll associate feminine with girl, that’s more difficult […] these things really hurt me, but others, you know, they can’t understand that, like, how we really feel.”*

(Tina, 17-year-old non-binary student)*

Inappropriate cis-normative comments and remarks made by teachers and classmates, even if made unintentionally, had a negative impact on TNB participants’ well-being. 

Restrictive cultural representations of gender are marginalizing for TNB youths. These manifest in many spaces at school, but particularly in gendered spaces, such as bathrooms and locker rooms. Gendered locations are an important dimension for validation of gender identity, but often standardize a binary vision of gender. These spaces were often perceived as uncomfortable by youth:

“[…] Having to choose every time where I feel the most comfortable and like, I don’t want to make other people uncomfortable either […]” *

(Tina, 17-year-old non-binary student)

In most cases, TNB students were not consulted about their preference regarding the use of gendered spaces or other trans-inclusive accommodations and had to endure measures that could be as discriminatory, or even more discriminatory, as no accommodation at all. Some youth could use the bathroom of their choice, for example a gender-neutral bathroom, but this preferred bathroom was sometimes far out of their way, which also can cause problems:

‘’I had asked not to have to go to a boy or a girl bathroom, so they told me to go... to go to a toilet that is really really far!’’*

(Max, 16-year-old, non-binary student)

However, having access to gender neutral bathrooms was not a solution that was desired by all. For example, Eric, a 15-year-old trans boy explained that he going to the boys’ bathroom until the school director realized that he was trans, and then forced him to go to a ‘mixed’ bathroom and changing room despite him not wanting to. This had negative consequences on his wellbeing.:

*“Interviewer: At the beginning, you were going to change in the guys’ locker room but after, he told you to go in the mixed locker room* *?*

*Participant: In the mixed toilets.*

*Interviewer: In the mixed toilets?*

*Participant, Yeah.*

*Interviewer: Ok. Ok... How did you feel about all that?*

*Participant: Well badly. Because, you know …I want to-I want to go to the guys’ bathrooms, to the guys’ locker rooms.*

*Interviewer: What effect did it have on you?*

*Participant: Well that hurt me.”*

(Eric, 15-year-old, trans boy student)

Therefore, the lack of inclusion of TNB students in school decision processes about gender diversity was perceived as a form of resistance against supporting their identity and was experienced negatively. Max, a 16-years-old, non-binary student, explained how difficult it was for them, to have their chosen name used at their school because of the teachers and principals’ attitude of cisnormativity which treats trans identities as needing justification to be valid and to benefit from accommodation:

“Just for teachers to use it [chosen name] it was a big deal […] they asked me for a gender dysphoria diagnosis report and proofs that I was going to take hormones and everything when it shouldn’t even be necessary.” *

(Max, 16-years-old non-binary student)

Unjustified delays, discrepancies, or inequities in the attribution of accommodations were also cited as examples by participants as ways to resist supporting trans-inclusive rights.

Some were also concerned about the way their personal information was treated by the school system, and about possible breaches in confidentiality. Administrative documents, containing students’ sex and birth name (i.e., school documents or identification cards) were a major concern for confidentiality:

“It’s really hard to show my papers to others, like people I just met. They have to check my deadname. For me, my deadname is something really private […].” *

(Tina, 17-year-old non-binary student)

Students often need to show these documents in schools, forcing unwanted disclosure of one’s gender identity and, thus, undermining TNB students’ privacy. 

Yet, schools that implemented mechanisms to protect youth privacy and were able to maintain confidentiality of information were identified as a protective factor allowing participants to feel safe in their school. 

For example, Axel, a 15-year-old trans male, explains that despite his name not being changed legally, the school list shows his two names, so people call him correctly, which is in his experience, very helpful.

“I am in a school which is quite organized too... I was the first [trans youth], but they are really open, so it helps […] [I had to persevere] but they saw that this was important. They said they would take steps [to adapt the school], and they did it with me... they have always been there to help me.’’*

(Axel, 15-year-old, trans male student)

#### 3.1.2. Direct Attitudes and Behaviours of Adults in Positions of Authority

Participants clearly expressed that school staff’s attitudes and behaviours, whether they were teachers, school personnel or administrators had an impact on their experience of gender identity validation. 

Staff support was identified as a protective factor for TNB students’ well-being. Conversely, a lack of support by school staff was harmful for TNB students’ well-being:

“[…] the first person I told [about my gender identity] was the social worker at my school, and since, she has always been super supportive […] she’s helped me a lot… it’s like a super important relationship for me.” *

(Olivier, 17-year-old trans male student)

“Teachers have a closed mind, and they really influence the students a lot, so, I don’t know, if at least teachers were there to support me it would’ve been that already, like something, but not even.” *

(Tina, 17-year-old non-binary student)

Indifference toward TNB students’ needs, as manifested by teachers and school personnel, was also seen as problematic by study participants. Students described feeling that school personnel did not seem concerned about their needs:

“Let’s say I was talking about my needs and all, and school personnel, in moments like these are just like: ‘Ok, yeah, but it’s your problem’ like they don’t even care.”*

(Max, 16-year-old non-binary student)

“[My teacher said:] Yeah OK, but you’re just one student and there are 30 other students in the class.”*

(Skyler, 16-year-old non-binary student)

Feeling ignored was reported as an important barrier for the inclusion of TNB students at school. Participants also felt ignored by staff who misused their preferred name and pronouns. Conversely, when staff used the correct name and pronouns, participants felt validated.

Teachers, principals, and school personnel’s incredulity about students’ gender identity was identified as a major obstacle by many students. Skyler, a 16-year-old, non-binary youth, explained that they were pressed with questions—sometimes irrelevant and intrusive—about their gender identity by their school vice-principal:

“I had to explain almost everything to the vice-principal and then she asked me weird questions.” *

(Skyler, 16-year-old non-binary student)

Finally, some schools and adults adopted a more repressive approach. Zack, a 17-year-old non-binary student, provided examples of situations where they lost points or failed school assignments for expressing their gender identity:

*“I actually failed a French writing assignment in grade 9 because I wrote using male pronouns.”*

(Zack, 17-year-old non-binary student)

The lack of acknowledgment and denial of student gender diversity by an adult in a position of authority was a recurrent theme throughout the interviews, and participants detailed these situations extensively. 

#### 3.1.3. Peer Attitudes and Behaviours toward Gender Diversity and TNB Students

Acceptance and acknowledgment of gender diversity by classmates was also an important component of TNB youths’ well-being. Students who reported a higher level of well-being at school felt that their gender identity was acknowledged and supported by their peers. Remi, a 17-year-old non-binary student, explained how they felt their affirmed identity was immediately accepted by their high school friends, because they spontaneously adopted their right name and pronouns:

“[My friends], they accepted my transition really well and they switched my pronouns and my name pretty much on the spot, so that was really cool. And it had a really positive effect on me.” *

(Remi, a 17-year-old non-binary student)

On the contrary, the misuse or refusal to use preferred names and pronouns by peers was detrimental to several youth who explained they felt invalidated and that it had a negative impact on their well-being. Discussing how peers refused to affirm their gender identity through their name and pronoun, this youth show how their mental health deteriorated:

“Because, you know, before people started using the right pronouns and the right name with me, I ended up on the psychiatry unit like three times.”*

(Arthur, 17-year-old non-binary student)

Student Associations and groups, such as Gay-Straight Alliances (GSAs) were a major source of peer support. Marlie, a 15-year-old and trans girl, explained how positive her experience at school was:

*“At my school, we have a GSA. […] So, all of the LGBTQ kids, at our school, we, like, go there.”*

The presence of LGBTQ+ organized activities at school provided youth with support, and a sense of belonging. Some youth also reported being involved with arts or other shared interest clubs, which was helpful. However, not all youth had positive experiences with their peers.

Some participants experienced indifference which could be either positive, or negative. For some, indifference was a sign of tolerance, or even sometimes, acceptance:

*“And people were like, ‘Oh yeah. She’s trans, or whatever. And so, no one really cares that much, which is good”*

(Marlie, 15-year-old trans girl student)

“It’s ok ‘cause people, they don’t care, but at the same time they accept it.”*

(Eric, 15 years-old trans male student)

Some participants described explicitly that they felt their identity was disrespected by other students. One manifestation of this lack of acceptance was the passive rejection experienced by some of them, which lead participants to feel isolated and lonely. This passive form of rejection was described as an insidious process:

“And you know, even my friends at school, I am sitting at the table with them for lunch and I feel like I’m sitting with strangers, you know. I feel alone surrounded by people, so well”. *

(Arthur, 17-year-old non-binary student)

Others described situations where they were excluded from school social interactions, deliberately set aside by their peers and friends or even bullied after revealing their gender identity or coming-out. Bullying was mostly perpetrated through transphobic comments, invasion of privacy and deliberate misuse of preferred names and pronouns. Marlie, a 15-year-old trans girl, detailed how a friend rejected her after her coming-out, and then bullied her by deliberately disclosing her identity to others without her consent:

*“ […] And one of them was, like, one of my ex-friends, which is not that great. […] And he was making fun of me for it and stuff, and telling other people about that, so that’s not cool.”*

(Marlie, a 15-year-old trans girl student)

Being victimized and rejected at school, as reported by Marlie, has strong negative consequences on TNB youths’ well-being. 

### 3.2. Theme 2: Strategies Used by TNB Students to Face Adversity

Numerous strategies used by TNB students to face adversity at school were identified in the transcripts of the open interviews with study participants. 

Youth participants described several strategies to face their peers’ wrongful behaviours. Among these strategies, some TNB youths surrounded themselves with allies as a strategy to face bullies and oppressors. Olivier explained how having such close friends at school kept him from being bullied after he disclosed his gender identity:

“For sure, if people had comments to make, I mean, I really have a lot of friends and I know that my friends are there to defend me […].” *

(Olivier, 17-year-old trans male student)

When victimization and bullying were inevitable, some participants reported actively avoiding bullies by avoiding certain places at school or social interactions. Other participants described intentionally steering clear of unaccepting people and deliberately ignored oppressing situations or persons. Like Axel, a 15-year-old trans male, who described how he reacted when he faced someone’s contempt towards him:

“I don’t react badly–these people they don’t accept me the way I am. It’s not worth it to stay with them […].” *

(Axel, 15-year-old trans male student)

Strategies were used to face issues with peers but also adults in positions of authority. Among these, to educate, to make people aware and to advocate for TNB rights and realities were the main strategies employed. As explained earlier by Skyler who had to answer intrusive questions about their gender identity, a lack of understanding of gender diversity appeared to contribute to school staff’s resistance to offer an inclusive environment. Accordingly, educating and raising awareness about gender diversity, and self-advocacy were key strategies used by students to face invalidation and other obstacles. However, as explained by Skyler, these strategies were not always protective of TNB youths’ well-being, as they were often imposed on them:

“[…] we get the impression that we have to explain everything for them to feel comfortable, or for them to agree to [offer a trans-inclusive accommodation], but at the same time we don’t feel like doing it sometimes.” *

(Skyler, 16-year-old non-binary student)

Others used compromise as a strategy to face school personnel and principals’ resistance toward providing a trans-inclusive environment. Coralie, a 15-year-old trans girl, used this strategy when she asked to use the girls’ bathroom and was denied this right by the school’s administration. Coralie settled on using the gender-neutral teachers’ bathroom. She commented:

“[…] At least we were able to meet halfway.”*

(Coralie, a 15-year-old trans girl student)

All twelve TNB students publicly disclosed their gender identity during high school. However, some of them had to change schools- whether because they graduated or because they moved to a different area. Those subjects did not necessarily redisclose their non-cisgender identity at their new school. Depending on the context, TNB chose to either disclose, partially disclose or not disclose their gender identity to others. Their choice was often associated with a sense of safety in the new environment, again showing the importance of school climate and culture (discussed in Theme 1). As Skyler illustrates:

“Sometimes I hide my identity […] let’s say I’m with an adult that might be in a position of authority or if I’m in an alley and it’s nighttime and I don’t feel like getting into trouble.”*

(Skyler, 16-year-old non-binary student)

Others, like Remi, sometimes chose to partially disclose their gender identity to avoid uncomfortable situations:

“Even when I tell people I’m trans, I only say that I am a guy, I don’t say that I’m non-binary because it’s complicated and I don’t feel like explaining”*.

(Remi, a 17-year-old non-binary student)

Maintaining control over the privacy of their gender identity was important for participants. Thus, the choice of strategy often depends on the specific context and circumstances faced by TNB youths. 

## 4. Discussion

Our data highlight some dimensions of school culture and climate around gender, and the impact they had on youth, especially regarding their experiences of validation or invalidation of their identity, such as the importance of supportive school associations or personnel [30]. Ullman [31] explains that gender climate is observable through various dimensions such as organisational, educational, and interpersonal cultures that promote or, on the contrary, hinder gender or sexual orientation among youth in all their diversity. In our study, through behaviours and attitudes of teachers, school personnel and peers notably, it was possible to illustrate that they can positively or negatively impact TNB youth experiences. As such, our study contributes to improving understanding of how school climate can impact TNB youth. While intercultural belonging or equity at school [32] as well as gender climate with regard to pupils with same-sex attraction [33] have been well documented and have been found to affect pupils’ experiences [11] gender climate specifically as experienced by TNB youth in schools remains less documented [33]. Our study shows how rightful acknowledgment of gender identity, when integrated through school climate, may contribute to providing a safe and inclusive environment for TNB students. Study participants also described many elements of their school climate as unsupportive. As previously described in the literature, prejudices, misconceptions, and non-inclusivity of gender diversity are often anchored into school culture [16] and cisnormativity strongly taints the public education system often pushing TNB identities into invisibility [34,35]. The hostile environment observed in some schools created by passive and active rejection, bullying, gendered spaces, and refusal to acknowledge gender diversity can thus contribute to psychological distress among gender minority youth [5,16,30,36].

In our research, participants illustrated many situations where their identity was either validated and/or supported, or, conversely, ignored, rejected and, even, repressed. Youth were also victims of discrimination, which took many forms. As identified in this and other cross-sectional studies, discrimination from peers led TNB youth to experience rejection, victimization, and bullying [2,9,13,20]. Interestingly, our results suggest that invalidating attitudes and behaviours of adults in positions of authority may have an even stronger impact on TNB students’ well-being. In fact, school personnel, teachers and principals’ attitudes and behaviours were mentioned more often and with more depth and details than peers’ attitudes and behaviours.

Similar observations have been made in previous studies that have explored more widely how teachers, principals and school personnel can influence TNB students’ lives, both negatively and positively [9,21,37,38,39]. Our findings are consistent with Singh [39] who has identified that many non-parental adults’ behaviours, such as lack of support, inaction in the context of transphobic peer bullying, repressive measures, and disregard of students’ needs are detrimental for TNB youths’ well-being. Conversely, the use of preferred name and pronouns [40] and respect of confidentiality during interactions with adults [37] were identified as protective and supportive factors in both our study and other studies. Overall, findings from our analysis highlight the importance of an adequate, genuine, and unconditional acknowledgement of TNB students’ gender identity at school through the development of a more inclusive gender climate. 

Our study has also allowed to identify strategies used by TNB youth when facing adversity, such as to educate, to compromise, and to advocate. These are varied and contextually driven [26]. While our sample was mostly homogenous in terms of cultural identity, the data on school experience allowed us to observe that youth use a wide range of strategies that are highly context dependent. In fact, while some youth may challenge or confront, others may use avoidance and hiding, or comprising.

Previous research has shown that TNB youth will alternate strategies of resistance depending on the context and may sometimes avoid them altogether if they feel unsafe [41]. While our sample was too small to fully explore how strategies are used in specific school contexts, our data suggest that increasing safety and inclusiveness of school environments can facilitate youth feeling of recognition and safety, and ultimately wellbeing. Of note, concealing one’s gender identity is a strategy of resistance and protection for some youth, but it may not, in the long term, prevent them from benefiting from full recognition, and may contribute to maintaining them in a context of minority stress.

### 4.1. Implication for Practice

Given individual differences and specific needs for each student, TNB youth should be involved in decision-making processes at school. In our research, Axel, for example, explained how it was helpful to have the school adapt to trans pupils, and doing it *with him.* Conversely, Eric’s story regarding bathroom use illustrates how not listening to a young person’s needs may have detrimental consequences on them. No assumptions should be made regarding how to adequately address their needs as each TNB person and experience are different [34,42] as reflected by the diversity of perspectives highlighted in our study. However, as mentioned by most, if not all, of the interviewed youths and by other authors, gender diversity often only becomes a matter of interest when a student discloses their TNB gender identity [43,44]. Thus, even if the individual needs of some gender minority students are considered in a growing number of schools, core obstacles to gender diversity acknowledgment, such as cisnormativity and transphobia, are rarely addressed [43]. As many children begin to explore their gender identity, school climate should be open to gender diversity, even in the absence of other students who may have already disclosed a TNB gender identity [37,42,44]. Even in jurisdictions where legal protections are in place, discrimination, including from those in positions of authority is still being experienced by youth, and school gender climate continues, in many places, to be unsupportive of TNB youth. To address this, schools should be forthcoming and prepare themselves to be inclusive of trans and non-binary youth, even if no gender diverse youth have yet expressed any particular needs. To do so, schools can draw on existing documents that promote good practices for the inclusion of trans and non-binary youth in schools (For example, schools could refer to the document *Measures for Openness to and support for trans and non-binary students: a guide for educational institution*s, for guidance in making adaptations to support TNB students in schools. See: https://familleslgbt.org/app/uploads/2022/03/Measures-for-openness-to-and-support-for-trans-and-non-binary-students.pdf, accessed on 8 September 2022). This could be done by schools inviting trans youth support organizations to provide education on gender identity and the needs of trans youth to all pupils, and plan specific training for school staff. As such, when a trans or non-binary youth enrolls in their school, they would be better prepared to meet their needs, and would already have access to tools and strategies to respect and affirm their students’ gender identity. By inviting trans youth support organizations to talk about trans youth experiences, they would sensitize all students to the possibility that one of their friends may one day affirm a gender-diverse identity and would facilitate the development of a more positive gender climate.

### 4.2. Study Limitations

There are some limitations to the current study. We only selected 14 to 17-year-old teenagers to examine their specific experience in high school settings, resulting in a sample of only twelve participants. Given the limited size of our sample and the complexity of the issues at stake, it is unlikely that the present study achieves data saturation. Additionally, our sample was not culturally and geographically diversified, even though the sample of the overarching study was. Thus, important topics, such as intersectionality in school settings and differences between rural and metropolitan schools, may not have been appropriately captured in our data. Finally, interview questionnaires were not built specifically to assess the complete school experience of TNB youths, but rather to explore TNB youths’ well-being in general. Thus, this study is not fully representative of TNB students’ lived experiences at school and some topics might not have been explored. However, this specific limitation also represents a strength of our study given that TNB youths could spontaneously express which factors they felt had the strongest impact on their well-being. The fact that school-related aspects were discussed so thoroughly speaks strongly to the magnitude of the impact of school factors on TNB youths’ well-being.

## 5. Conclusions

Our study reveals that openness, validation, and support of gender diversity at school can positively affect TNB youths’ well-being. Conversely, various forms of non-recognition of gender identity, victimization and bullying towards TNB youths impede their wellbeing and should not be tolerated at school. Schools should proactively ensure that they put in place measures that will facilitate the inclusion of gender diverse young people and adopt strategies that respect and affirm youth gender identities. Such measures should pay attention to confidentiality of information to protect students’ privacy regarding their gender identity. While these themes have already been explored in the literature, our qualitative data allowed us to illustrate how they are experienced by young people in the school setting. While our study methodology was not meant to identify all possible adaptations and improvements that could be made to support TNB youth in the school setting, our data nevertheless demonstrate the importance of improving schools’ gender climate. Peer, teacher, school personnel, and principal support is essential and contributes to TNB students’ well-being. While TNB youths should be included in decisions that concern them, schools have the responsibility and potential to make every one of their students, regardless of their gender identity, feel safe and included.

## Data Availability

Not applicable.
